# Protocol: Interventions to Prevent Cognitive and Behavioural Violent Radicalisation: A Systematic Review and Multilevel Meta‐Analysis: A Systematic Review

**DOI:** 10.1002/cl2.70058

**Published:** 2025-08-10

**Authors:** Sara Valdebenito, Manuel Eisner

**Affiliations:** ^1^ Institute of Criminology University of Cambridge Cambridge UK; ^2^ Institute of Criminology Wolfson Professor of Criminology Cambridge UK

## Abstract

This review aims to systematically evaluate the available evidence on the effectiveness of various interventions aimed at reducing violent radicalisation and terrorism. Quantitative analysis will be employed to assess the overall impact and identify the factors that influence it. The key research questions are: (1) What interventions are successful (A successful intervention will be defined as one that effectively reduces cognitive radicalisation (e.g., extremist beliefs or ideological commitment), reduces behavioural radicalisation (e.g., intentions or actions related to violent extremism), prevents at‐risk individuals from becoming radicalised (counter‐radicalisation), or facilitates the disengagement and de‐radicalisation of individuals already involved in extremist activities or networks) in preventing or reducing violent radicalisation and terrorism? (2) Are certain preventive strategies more effective than others? (3) How do participants' characteristics influence programme outcomes? (4) How do the features of interventions, their implementation, and methodologies impact the effectiveness of terrorism prevention? The review will conduct analyses considering participant characteristics (e.g., age, gender, risk level), intervention components (e.g., theoretical foundations, theory of change), implementation (e.g., facilitator training, dosage), and methodological aspects (e.g., study design). The goal is to identify the most effective interventions, the target groups they work for, and the conditions under which they are most successful.

## 1 Background

### 1.1 The Problem, Condition, or Issue

Violent radicalisation is a complex sociopolitical process through which individuals or groups adopt extreme ideological positions that justify the use of violence to achieve political, religious, or social objectives (Hafez and Mullins 2015). It constitutes a significant global challenge, contributing to the prevalence of terrorism, extremism, political violence, and societal destabilization. The multifaceted consequences of violent radicalisation—ranging from significant human and economic losses (Bardwell and Iqbal 2021) to erosion of societal cohesion and security (Ben‐Porath 2023; Rivers 2018)—highlight its urgency as a focal issue for policymakers, practitioners, and researchers. Addressing violent radicalisation effectively requires not only understanding its underlying mechanisms but also identifying evidence‐based interventions capable of mitigating its impact.

The process of violent radicalisation is not linear nor homogenous, but rather emerges from the interplay of individual, social, and structural factors (Amit and Kafy 2022; Feddes et al. 2023; Hassan et al. 2021). At the individual level, vulnerabilities such as identity crises, perceived injustice, moral disengagement (Aly et al. 2014; Feddes et al. 2015), marginalisation (Enders et al. 2016), or unmet psychosocial needs intersect with broader sociopolitical dynamics, including economic deprivation, systemic discrimination, or exposure to extremist ideologies (Copeland and Marsden 2020).

Social influences, including peer networks, community dynamics, and online platforms, play a central role in amplifying and disseminating radical narratives (Moyano et al. 2024). Moreover, structural conditions—such as weak governance, geopolitical conflicts, and political instability—create enabling environments for violent radicalisation to emerge and proliferate (Coggins 2015; Feddes et al. 2023). The heterogeneity of these drivers complicates the identification of universal predictors or pathways to radicalisation, thus presenting significant challenges to intervention design. The lack of clarity regarding what constitutes success or effectiveness in violent radicalisation interventions further compounds the challenges in designing and evaluating these programes (Glazzard 2025).

Despite the sustained interest among policymakers and scholars in addressing radicalisation and terrorism, a universally accepted definition of these terms remains elusive (Enders et al. 2011; Ramsay 2015), leading to the interchangeable use of terms like terrorism, extremism, and radicalisation. However, the synonymous use of these terms offers little clarity to the ambiguity they seek to simplify because they represent different facets of a complex issue.

A critical framework that enhances understanding of violent radicalisation is the *Two Pyramid Model* (McCauley and Moskalenko 2017), which differentiates between ideological and behavioural dimensions of radicalisation. This model posits two interrelated pyramids:

1. *The Opinion Pyramid:* Represents the spectrum of ideological positions, ranging from neutral or moderate beliefs at the base to extremist ideologies that justify violence at the apex.
2. *The Action Pyramid:* Depicts the spectrum of behaviours, ranging from lawful and passive activities at the base to active involvement in violent acts at the apex.

The model emphasises that not all individuals who adopt radical ideologies progress to violent behaviours and that some individuals may engage in violent actions without firmly adhering to extremist ideologies. While extremist beliefs (cognitive violent radicalisation) are a risk factor, they do not inherently result in violent actions (behavioural violent radicalisation). Understanding this distinction is crucial for tailoring interventions, as some programmes may focus on preventing the spread of extremist beliefs (e.g., a decline in the belief that violence is a legitimate means to achieve political, religious, or social goals, improved capacity to resist recruitment efforts and extremist narratives, confidence in democratic institutions, legal systems, and non‐violent methods of addressing grievances) while others aim to disrupt behavioural pathways to violence (e.g., disrupt recruiting of individuals to extremist causes or groups, interrupt participation in physical violence, attacks, or terrorism‐related activities, disrupt disseminating extremist narratives). By delineating the distinction between belief and behaviour, the Two Pyramid Model offers a nuanced framework to investigate the mechanisms driving radicalisation and can support the development of targeted interventions tailored to the specific stages of the process. This approach also underscores the importance of designing interventions that address the ideological and behavioural aspects of radicalisation separately, depending on their interaction and manifestation in different contexts.

Despite significant investments in interventions to address violent radicalisation—ranging from counter‐narrative campaigns and community engagement initiatives to rehabilitation programmes for at‐risk individuals—the evidence remains elusive. Studies often suffer from methodological limitations, including small sample sizes, lack of control groups, and short follow‐up periods, thereby limiting their generalisability and applicability across contexts (Bellasio et al. 2018; Brouillette‐Alarie et al. 2022). Furthermore, many interventions operate in context‐specific environments, with cultural, social, and political differences influencing their efficacy. For example, deradicalization programmes targeting jihadist radicalisation in conflict zones may not be transferrable to efforts aimed at addressing far‐right extremism in Western democracies (Vidino 2010). Compounding this challenge is the risk of unintended consequences, such as stigmatisation of certain communities or the erosion of trust between marginalised populations and state institutions (Brouillette‐Alarie et al. 2022).

To address these gaps, a systematic evaluation of interventions targeting violent radicalisation is critical. This review seeks to synthesise the current body of evidence, identifying effective strategies while highlighting methodological strengths and limitations within the literature. In addition, the review will assess contextual factors that mediate the success or failure of interventions, providing a comprehensive understanding of what works, for whom, and under what circumstances. By incorporating conceptual frameworks like the Two Pyramid Model, the review will contextualise the findings within the broader spectrum of radicalisation processes, offering actionable insights for policymakers, practitioners, and researchers. Ultimately, addressing violent radicalisation requires not only robust, evidence‐based interventions but also a coordinated effort to address its root causes and foster social resilience against extremist ideologies.

### 1.2 The Intervention: How the Intervention Might Work

A logic model is a structured framework that represents the relationships between an intervention's inputs, activities, outputs, and intended outcomes, providing a clear roadmap for understanding how an intervention is expected to achieve its goals (Hassan et al. 2021).

The logic model of the present systematic review and meta‐analysis will involve the differentiation between cognitive and behavioural outcomes (McCauley and Moskalenko 2017) as well as the public health prevention approach at both the secondary and tertiary levels (Harris‐Hogan et al. 2016; Hassan et al. 2021). *Secondary prevention* focuses on individuals or groups at higher risk of violent radicalisation. These interventions are based on reliable assessments indicating vulnerability to extremist ideologies or behaviours. The objective is to intervene before any violent action or firm attachment to extremist ideologies occurs (Brouillette‐Alarie et al. 2022). Programmes in this category might be personalised, addressing specific vulnerabilities that could lead to violent radicalisation (Feddes et al. 2015). These programmes—often implemented as part of broader Countering Violent Extremism (CVE) efforts—include mentorship, community engagement, and educational initiatives aimed at building resilience and critical thinking skills. By providing alternative narratives that counteract the appeal of extremist ideologies, secondary prevention seeks to intervene at an early stage, mitigating risk factors before individuals adopt violent ideologies or engage in extremist behaviours while simultaneously enhancing protective factors that prevent violent radicalisation and associated violent behaviours. *Tertiary prevention* targets individuals who have already shown signs of violent radicalisation or participated in extremist activities. The focus is on disengagement, de‐radicalisation, and reintegration into society (Brouillette‐Alarie et al. 2022). These programmes work with individuals to renounce violence and facilitate ideological shifts away from extremism (e.g., Webber et al. 2018). Tertiary interventions are crucial for breaking the cycle of violence and helping individuals reclaim a non‐violent identity within their communities. This level is often the most challenging, requiring tailored approaches to address deep‐seated beliefs and experiences that lead individuals toward violent radicalisation (Harris‐Hogan et al. 2016). Using a logic model to evaluate tertiary interventions enables the systematic identification of the processes and mechanisms required to address entrenched ideologies and behaviours. It also ensures that the outcomes—such as behavioural disengagement and successful societal reintegration—are clearly linked to intervention activities and measurable metrics.

### 1.3 Why It Is Important to Do This Review

Despite the growing interest in preventing violent radicalisation and terrorism, the evidence supporting the effectiveness of various interventions remains limited. While numerous programmes and strategies have been developed and implemented across different contexts, rigorous evaluations and systematic analyses of these interventions are sparse (Nehlsen et al. 2020). Current evaluations are often criticised for their insufficient standards and lack of rigour (Bellasio et al. 2018; Brouillette‐Alarie et al. 2022; Mazerolle et al. 2021), with low internal validity identified as a significant obstacle to advancing the field (Jugl et al. 2020, 37). This gap in the literature presents a significant challenge for policymakers and practitioners who seek to implement evidence‐based approaches to counter violent extremism. When it comes to evidence synthesis, such as systematic reviews, many of the published findings focus on narratives rather than quantitative analysis of impact (Amit and Kafy 2022; Pistone et al. 2019; Stephens et al. 2021). Consequently, there is an urgent need for more comprehensive and robust research to assess the impact of prevention programmes and to identify best practices in the field. By addressing these gaps, future research can contribute to a more nuanced understanding of what works in preventing violent radicalisation, thereby enhancing the effectiveness of efforts to safeguard communities and promote social cohesion.

### 1.4 Previous Reviews and Meta‐Analyses

The Campbell CVE project has contributed valuable insights into the issues of terrorism and violent extremism. A series of Campbell Systematic Reviews have thoroughly examined the literature on the factors driving violent radicalisation/extremism and terrorism. These reviews have focused on a wide range of contributing factors, including psychological traits (Carthy et al. 2020; Sarma et al. 2022), family influences (Zych and Nasaescu 2022), sociodemographic variables, experiences of deprivation, connections with radicalised peers, tendencies toward risk‐seeking behaviour (Wolfowicz et al. 2021), and media exposure (Wolfowicz et al. 2022), among others.

In terms of evaluating interventions, some previous reviews have centred on approaches designed to curb violent radicalisation, violent extremism, and terrorism. These reviews have explored various methodologies, including case management interventions (Lewis et al. 2023), the use of counter‐narratives (Carthy et al. 2020), as well as the effectiveness of multi‐agency collaborations (as examined in a single study) (Mazerolle et al. 2021) and the impact of police‐community relations (analysed in another single study) (Mazerolle et al. 2020). Additionally, one study outside the Campbell Systematic Reviews assessed the role of educational interventions, though it did not involve a meta‐analysis (Sjøen and Jore 2019). Despite identifying several narrative reviews aimed at reducing violent radicalisation and violent extremism (Brouillette‐Alarie et al. 2022; Parker and Lindekilde 2020; Sydes et al. 2023), these reviews often lacked statistical data extraction, and their methodological rigour did not consistently align with the standards established by the Campbell Systematic Reviews. To ensure a comprehensive assessment, our review will also incorporate recent interventions, building on the insights from previous studies. Furthermore, we will employ a multilevel meta‐analysis model to synthesise the effects of these interventions.

## 2 Objectives

This review aims to systematically evaluate the available evidence on the effectiveness of various interventions aimed at reducing violent radicalisation and terrorism. Quantitative analysis will be employed to assess the overall impact and identify the factors that influence it.

The key research questions are:

- What interventions are successful (A successful intervention will be defined as one that effectively reduces cognitive radicalisation (e.g., extremist beliefs or ideological commitment), reduces behavioural radicalisation (e.g., intentions or actions related to violent extremism), prevents at‐risk individuals from becoming radicalised (counter‐radicalisation), or facilitates the disengagement and de‐radicalisation of individuals already involved in extremist activities or networks) in preventing or reducing violent radicalisation and terrorism?
- Are certain preventive strategies more effective than others?
- How do participants' characteristics influence programme outcomes?
- How do the features of interventions, their implementation, and methodologies impact the effectiveness of terrorism prevention?

The review will conduct analyses considering participant characteristics (e.g., age, gender, risk level), intervention components (e.g., theoretical foundations, theory of change), implementation (e.g., facilitator training, dosage), and methodological aspects (e.g., study design). The goal is to identify the most effective interventions, the target groups they work for, and the conditions under which they are most successful.

## 3 Methodology

### 3.1 Criteria for Considering Studies for This Review

#### 3.1.1 Types of Studies

This systematic review will include studies based on experimental and quasi‐experimental designs (QEDs) (Shadish et al. 2002). Randomised controlled trials (RCTs) must include at least one group receiving the intervention (experimental group) and a comparison group (control group), with participants randomly assigned to each condition. The control group may receive no intervention, a standard intervention, a waitlist condition, or a placebo intervention.

Eligible RCT designs may include:

- Individually RCTs;
- Cluster RCTs;
- Waitlist‐Controlled Randomised Trials.

Clustered trials will be considered, with appropriate adjustments made for the amalgamation of individual and clustered data (see Section 3.3.6).

QEDs eligible for inclusion must, at a minimum, include pre‐ and post‐intervention assessments to evaluate changes that may be attributable to the intervention.

Eligible QED designs may include:

- Non‐randomised Control Trial (compared between treatment and control groups non‐randomised but equivalent based on the specific variable).
- Non‐Equivalent Groups Design (compares outcomes between treatment and control groups without random assignment and without equivalent groups, risking selection bias).
- Propensity Score Matching (reduces this bias by matching individuals with similar characteristics across groups).
- Interrupted Time Series Design (analyses trends over time to detect changes following an intervention).
- Regression Discontinuity Design (estimates effects at a cutoff point on a continuous assignment variable).
- Before‐and‐After Design (measures outcomes before and after an intervention in the same group, without a control group).

In our final review, we will separately analyse and present findings from RCTs and different types of QEDs to ensure transparency in methodological distinctions.

The following study types will be excluded from this review:

- Qualitative studies;
- Purely observational studies (e.g., correlational studies without intervention);
- Cross‐sectional surveys that do not assess change over time;
- Case studies or anecdotal reports.

#### 3.1.2 Types of Participants

The target population for studies included in this review is any young (i.e., older than 12 years of age) or adult individuals who are at risk of, or involved in, the process of cognitive or behavioural violent radicalisation or acts defined as terrorist. Therefore, included individuals may be those at risk, who could potentially de‐escalate in opinion or action (secondary level of prevention), or those who have already been charged (e.g., individuals transitioning from prison to the community). We will not impose restrictions related to gender or ethnicity.

#### 3.1.3 Types of Interventions

The scope of this review includes interventions targeting individuals or groups at various stages of violent radicalisation, encompassing both those at risk of perpetrating violent acts and those already involved in extremist activities. The review will cover interventions implemented by individuals or teams across different sectors, including the criminal justice system (e.g., Sydes et al. 2023), educational institutions (e.g., Parker and Lindekilde 2020), psychosocial support frameworks (e.g., Jugl et al. 2020), and community‐based initiatives (e.g., Mazerolle et al. 2020). By incorporating diverse methodologies and targeting various populations, this review seeks to identify effective approaches to CVE and promoting social cohesion. The analysis will highlight the efficacy of these interventions in reducing the incidence of violent radicalisation and terrorist activities, thereby contributing to the development of evidence‐based policies and practices.

This review will exclude studies that focus on interventions aimed at supporting victims of terrorist violence. Specifically, it will not encompass programmes designed to aid children and families in communities affected by such acts, either in the immediate aftermath of incidents or afterward (e.g., Berger et al. 2007). Additionally, interventions targeted at providing support to second responders, including police officers, medical personnel, and firefighters, will also be outside the scope of our analysis, which is mainly focused on perpetrators. Furthermore, interventions related to government policy changes will not be included (e.g., LaFree and Freilich 2019). Situational prevention strategies, such as the implementation of metal detectors, walls, and street barriers, are likewise excluded from this review (Lum et al. 2006; Perry et al. 2017). Also, studies targeting counter‐narrative and online media campaigns will be excluded from the analysis (e.g., Carthy et al. 2020).

#### 3.1.4 Types of Outcome Measures

The review seeks to evaluate the impact of interventions on outcomes related to violent extremism, focusing on both cognitive and behavioural aspects. A conceptual framework distinguishing cognitive and behavioural dimensions of violent radicalisation is displayed in Table 1.

**Table 1:** Outcomes planned to be extracted from included studies.

Conceptual domain	Cognitive outcome	Behavioural outcome
Violent beliefs	− Support for violent radicalisation, violent acts, and conspiracy theories. − Belief in the legitimacy of violent actions and terrorism.	− Sharing of narratives supporting violent radicalisation. − Terrorism‐related acts, arrests, or probation breaches.
Critical thinking and resilience	− Ability to evaluate propaganda and resist manipulative messages. − Recognition of recruitment tactics. − Knowledge about radicalisation, intolerance, and fanaticism. − Confidence in addressing exposure to radicalisation.	− Participation in networks promoting violent radicalisation. − Rejection of radicalisation efforts. − Avoidance of actions that support violent groups.
Perspective‐taking and social identity	− Political tolerance and respect for differences. − Recognition of shared societal values and inclusion.	− Participation in community‐building initiatives. − Avoidance of hate‐driven actions. − Establishment of stronger social networks to resist radicalisation.
Trust and norms	− Confidence in democratic systems, political legitimacy, and civic values. − Cognitive alignment with societal laws and norms.	− Compliance with laws and regulations. − Engagement in non‐violent conflict resolution and civic responsibilities.
Pro‐social behaviours	− Violence as a legitimate means of addressing grievances. − Contributing positively to society.	− Non‐violent behaviours. − Participation in volunteering, mentoring, or civic activities to counter radicalisation. − Actions taken to address violent radicalisation.
Disengagement	− Detachment from ideologies of violent radicalisation. − Acceptance of societal norms and development of a non‐radicalised identity.	− End of roles in networks supporting violent radicalisation. − Reintegration into family, education, or employment. − Building support systems for sustained reintegration.
Long‐term disangegament of violent radicalisation	− Skills to avoid ideologies and networks promoting violent radicalisation. − Confidence in recognising and countering violent recruitment content.	− No presence of recruitment activities and/or extremist group involvement. − Continuous efforts to address the risks of violent radicalisation.

Table 2 presents an example of outcomes extracted from studies identified during our pilot searches. These initial findings help illustrate the types of outcomes and instruments that are being reported in the relevant literature.

**Table 2:** Example of outcomes extracted from studies detected in pilot searches.

Publication	Outcome measured	Instrument
Saleh et al. (2023)	− Improved ability and confidence in assessing manipulative WhatsApp messages − Improved ability in identifying vulnerability to extremist recruitment	Participants are presented with simulated WhatsApp messages and cases (vignettes).
Aryuni et al. (2024)	− Prosocial behaviour − Respect: respect for others, respect for differences, and respect for places and things − Knowledge about radicalisation: intolerance and fanaticism	− Prosocial Personality Battery by Penner et al. (1995) (30 items) − Respect scale (30 items) − Radical Knowledge Test (20 items)
Parker and Lindekilde (2020)	− Political tolerance − Political efficacy − Ability to recognise extremist recruitment tactics − Confidence in knowing what to do if exposed to extremism − Perceived legitimacy of violence	Paper and pencil questionnaire (18 items)
Sahgal and Kimaiyo (2020)	− Attitude towards violence − Self‐confidence − Support systems and diversity of networks − Risks of joining terrorist organisations − Actions taken to address VE	Not specified
Moyano et al. (2022)	− Moral disengagement − Support for political violence − Social risk network − Social support	− Bandura et al.'s (1996) three items − Bélanger et al.'s (2018) three items − Moyano et al.'s (2022) three items

#### 3.1.5 Duration of Follow‐up

The included studies may describe the measurement of outcomes at various time points, such as immediately after the intervention, 1 year later, and 2 years post‐intervention. This approach allows for the inclusion of different measures taken at distinct time intervals, providing a comprehensive understanding of the intervention's effects over time. The review will incorporate these time‐specific measures to evaluate changes in effect sizes across different time points, highlighting any temporal variations in the intervention's impact. If changes in effect sizes are observed, they will be explicitly reported and discussed to enhance the interpretation of the intervention's long‐term effectiveness.

#### 3.1.6 Types of Settings

We plan to examine interventions focused on individuals or groups, delivered by a variety of entities including criminal justice agencies (e.g., prisons, probation departments), psychosocial interventions, interventions in educational settings (e.g., schools, universities), interventions in mental health services and community organisations (e.g., community centres, refugee camps, asylum centres).

#### 3.1.7 Publications Status and Type of Publication

Eligible studies for our review include both published and unpublished reports. We will consider a variety of sources, such as journal articles, book chapters, government reports, and academic theses at the MSc and PhD levels.

#### 3.1.8 Language

Studies from any country and in any language are eligible for inclusion, provided that the title and abstract are available in English. The inclusion of non‐English studies will also depend on the availability of resources and translation services.

### 3.2 Search Methods for Identification of Studies

The review aims to gather a comprehensive set of empirical studies from various countries and databases, both published and unpublished. An exhaustive search will be conducted to minimise publication bias, using a specific set of keywords covering three main dimensions: outcomes, type of study, and interventions. Table 3 outlines the keywords for each dimension.

**Table 3:** Proposed search terms.

Dimensions	Keywords
Outcomes	4chan, 8chan, 8kun, al‐Qaeda, al‐Qaida, anarch*, bioterror*, Boko Haram, bomb, bombed, bomber*, bombing*, bombs, cyber terror*, cyber‐terror*, cyberterror*, Daesh, extremis*, extreme‐left, extreme‐right, extreme* violen*, fanatic*, far‐left, far‐right, foreign agent*, foreign fighter*, guerrilla*, Hamas, Hezbollah, hijack*, incel, incels, indoctrinat*, insurrection*, insurgent*, ISIL, Islamis*, jihad*, KKK, Ku Klux Klan, klans*, lethal, lone, loner*, martyr*, militant*, militia*, mujahideen*, neonazi*, neo‐nazi*, QAnon*, radical*, Salafi*, skyjack*, supremacis*, supremacy, sympathiser*, sympathizer*, Taliban, terror*, terroris*, violen*, zealot*.
Type of study	allocat*, assign*, control* experiment*, control group*, control* study, clinical trial*, doubl* blind*, doubl* mask*, experiment controls, match* group*, nonrandom*, non random*, non‐random*, open label, open‐label, placebo*, pre‐post, quasiexperiment*, quasi‐experiment*, quasirandom*, quasi‐random*, random*, RCT*, singl* blind*, tripl* blind*, trial*.
Interventions	alternative*, approach*, at risk, at‐risk, campaign*, communit*, counter*, de‐radical*, deradical*, desist*, dialog*, disengag*, diversion*, divert*, engage*, engaging, factor*, guidance, implement*, initiative*, interact*, intervene*, method*, mitigat*, model*, policy, policies, practice*, prevent*, program*, project*, recidivis*, reduc*, re‐enter*, reentry, re‐entry, rehab*, reintegrat*, re‐integrat*, risk manage*, scheme*, stop*, strateg*, treat*.

The search terms will be combined using Boolean operators (e.g., AND, OR, NOT), along with wildcards and truncation symbols. As each electronic database has distinct symbol requirements, search terms will be adapted accordingly. A detailed record of each search will be kept, noting keywords, combinations, search dates, sources (e.g., databases, reference lists), and the number of studies identified and retrieved.

The management of bibliographic references will be facilitated through Zotero, a robust tool for organising and citing sources in academic research. The systematic review process, including screening and data extraction, will be conducted using Covidence, a platform specifically designed to support systematic reviews (Babineau 2014).

#### 3.2.1 Electronic Searches

In this review, a comprehensive search strategy will be employed, drawing from a range of electronic databases. These will include both published sources (e.g., ISI Web of Knowledge, PsycINFO) and unpublished reports (e.g., Dissertation and Thesis Global) to ensure a thorough examination of the relevant literature. Table 4 displays the databases and platforms that will be explored in our electronic searches. An example of a full search syntax is provided in Appendix S3.

**Table 4:** Databases and corresponding platforms for the review.

Number	Database	Platform
1	APA PsycNFO	Ovid
2	APA PsycEXTRA	Ovid
3	APA PsycArticles	ProQuest
4	Applied Social Sciences Index & Abstracts (ASSIA)	ProQuest
5	Book Citation Index (BCI)	Web of Science
6	Dissertations & Theses Global	ProQuest
7	Educational Resources Information Centre (ERIC)	ProQuest
8	International Bibliography of the Social Sciences (IBSS)	ProQuest
9	Social Services Abstracts	ProQuest
10	Sociological Abstracts	ProQuest
11	Sociology Database	ProQuest
12	Conference Proceedings Citation Index ‐ Social Sciences & Humanities (CPCI‐SSH)	Web of Science
13	Emerging Sources Citation Index (ESCI)	Web of Science
14	Social Science Citation Index (SSCI)	Web of Science
15	Social Sciences & Humanities	ProQuest
16	Criminal Justice Abstracts	EBSCO
17	International Political Science Abstracts	EBSCO
18	Scopus	Elsevier
19	Australian Criminology Database (CINCH)	Informit

#### 3.2.2 Searching Other Resources

We will reach out to key authors in the field who can guide us to specific publications essential for the review's objectives. During the data extraction and coding phase for the included papers, we will also contact authors if the provided study details are incomplete or if further statistical information is required for calculating effect sizes.

To ensure a thorough and comprehensive review, we will conduct both forward and backward citation searches. Forward citation searching will be performed for all included papers to identify studies that have cited them (e.g., Google Scholar), while backward citation searching will involve reviewing the reference lists of included papers and relevant reviews to locate additional studies.

To ensure a comprehensive review, we will also conduct hand searches of specific journals that are relevant to the topic of violent extremism. This approach will help identify studies that may not have been captured through database searches, thereby enriching the review. Below is a proposed list of journals to be included in the hand search:

- *Behavioral Sciences of Terrorism & Political Aggression*
- *Journal of Threat Assessment and Management*
- *Behavioural Sciences and the Law*
- *Studies in Conflict & Terrorism*
- *Frontiers in Psychology*
- *Aggression and Violent Behaviour*
- *The Journal for Deradicalization*
- *Terrorism and Political Violence*
- *Perspectives on Terrorism*
- *Nordic Psychology*
- *Criminology and Public Policy*
- *Psychology, Crime & Law*

In addition, the search will encompass a thorough review of the websites of both national and international organisations that have contributed evidence on the topic of violent radicalisation and terrorism. Table 5 provides an overview of the selected resources.

**Table 5:** Websites of organisations planned to be explored.

Organisation	Address
Department of Homeland Security	www.dhs.gov/topics/preventing-terrorism
EUROPOL‐European Counterterrorism Centre (ECTC)	www.europol.europa.eu/publications-events
eGlobal Terrorism Research Centre (Monash University)	www.monash.edu/arts/social-sciences/gtrec
Triangle Centre on Terrorism and Homeland Security	www.tcths.sanford.duke.edu
Department of Homeland Security	www.dhs.gov/topics
Public Safety Canada	www.publicsafety.gc.ca/index-en.aspx
National Criminal Justice Reference Service	www.ojp.gov/ncjrs-virtual-library/about-ncjrs
National Consortium for the Study of Terrorism and Responses to Terrorism (START)	www.start.umd.edu
Global Centre on Cooperative Security	www.globalcenter.org
RAND Corporation	www.rand.org/topics/terrorism.html?content-type=research
Radicalisation Awareness Network (RAN)	www.ec.europa.eu/home-affairs/what-we-do/networks/radicalization_awareness_network_enRadicalizationResearchhttps://www.radicalizationresearch.org/

### 3.3 Data Collection and Analysis

#### 3.3.1 Description of Methods Used in Primary Research

The studies included in the systematic review will encompass a range of research designs aimed at evaluating the effectiveness of interventions designed to reduce cognitive and behavioural violent radicalisation (see Table 6 for more details). These studies are expected to include a variety of experimental approaches, such as RCTs and QEDs, with interventions targeting both secondary and tertiary prevention strategies. As observed in Table 5, the interventions to be assessed will range from online inoculation games and mentoring programmes to counselling, sports‐based activities, and other educational or experiential approaches.

**Table 6:** Description of potential studies to be included (aims, characteristics and results).

Study	Aim of the study	Sample	Type of intervention	Outcomes measured	Results
Field Experiment in Iraq (Saleh et al. 2023)	To assess the effectiveness of the Radicalise game in improving resistance to extremist manipulation	191 vulnerable youth in post‐ISIS Iraq	Short online inoculation game (Radicalise)	Ability and confidence in assessing extremist messages; ability to identify vulnerable individuals	Improved ability to spot manipulative messaging (*p* = 0.034); no effect on identifying vulnerable individuals (*p* = 0.896)
Danish Youths P/CVE Study (Parker and Lindekilde 2020)	To evaluate the use of former extremists in preventing violent extremism	1931 Danish youths	Mentoring by former extremists	Perceived legitimacy of political violence; political tolerance	Reduced legitimacy of political violence; slight decrease in political tolerance
Kenya Mentoring and Counselling Study (Sahgal and Kimaiyo 2020)	To evaluate a mentoring and counselling programme for at‐risk youth in reducing vulnerability to violent extremism	347 at‐risk youth in Kenya	Mentoring and counselling	Attitudes towards violence; social networks; awareness of VE risks and countering strategies	Improved knowledge of VE risks and strategies; mixed results on attitudes and social networks; stronger effects in employed and longer‐exposed groups
Sports‐Based Intervention Study in Spain (Moyano et al. 2022)	To evaluate the impact of a sports‐based intervention programme on preventing violent extremism	213	Sport‐based intervention	Needs, violent narratives, and social networks of participants	Improvement in social networks; varied effects on needs and narratives

The review will encompass studies conducted in diverse geographical settings, targeting vulnerable populations such as youth in post‐conflict regions (e.g., Iraq), at‐risk youth (e.g., Kenya and Denmark), specific at‐risk communities, and individuals with migrant backgrounds. Interventions may be delivered through different formats, including lectures, workshops, experiential pedagogies, action‐learning techniques, role‐playing, and DVD‐based group sessions. These interventions aim to address various goals, such as decreasing bias against out‐groups, increasing resilience to radicalisation, improving critical thinking, promoting self‐esteem, enhancing empathy, and fostering perspective‐taking skills.

The studies will vary in duration, with some interventions being short‐term, lasting a few hours or days, while others are longer‐term, spanning several weeks or months. They will also address different forms of extremism, including religious, political, and violent extremist ideologies. The sample sizes across the studies will differ, ranging from smaller groups (e.g., 191 participants in Iraq) to larger studies (e.g., 1931 Danish youths), providing insights into the scalability of interventions (see examples in Table 6).

In summary, the review will include a wide range of primary studies assessing the effectiveness of diverse intervention programmes aimed at reducing violent radicalisation. These studies will be evaluated for their design, population characteristics, intervention methods, and the outcomes they measure, providing a comprehensive analysis of the effectiveness of these interventions in addressing violent radicalisation across various settings with special emphasis on secondary and tertiary levels of prevention.

#### 3.3.2 Selection of Studies

Two trained coders will independently determine the inclusion or exclusion of studies, following the predefined criteria outlined in Section 3.1. Table 7 displays the criteria for inclusion and exclusion at this stage (full screening tool in Appendix S2).

**Table 7:** Criteria for inclusion and exclusion: Screening.

Criteria for inclusion
Type of study
− Uses an experimental or quasi‐experimental design to evaluate the impact of interventions aimed at tackling violent radicalisation (RCTs or QEDs)
Type of participants
− Young (i.e., older than 12 years of age) or adult individuals who are at risk of, or involved in, the process of cognitive or behavioural violent radicalisation or acts defined as terrorist
Type of interventions
− Is the intervention aimed at preventing violent radicalisation?
Type of outcome measures
− Reports cognitive or behavioural outcomes related to violent radicalisation
Publication status/type
− Published and unpublished manuscripts − Journal article, book chapter, government report, or academic thesis
Language
− Title and abstract available in English

#### 3.3.3 Data Extraction and Management

Two coders will extract data from each included study using the data collection instrument found in Appendix S1. This data will be entered into an electronic database to generate descriptive and inferential statistics. If discrepancies arise between the coders at any point in the process, the principal investigator will be involved in the decision‐making process. Discrepancies will be resolved by consensus, and a record of these disagreements and their resolutions will be maintained. Twenty percent of the articles excluded at this stage will be cross‐checked by a third member of the team to ensure the reliability and accuracy of the decision‐making process. The use of two independent coders aims to minimise bias and reduce the risk of errors. Consistency in the inclusion/exclusion process will be assessed by calculating Cohen's kappa. This statistical measure will be used to evaluate the level of agreement between reviewers, ensuring that the data are reliable and that the criteria for targeting manuscripts are consistent across the study.

For the purposes of this review, studies will be coded in terms of publication features (e.g., author, year of publication, language), methodology (e.g., research design, sampling methods, attrition), participants (e.g., age, ethnicity, gender), characteristics of the intervention (e.g., setting, doses, training), role of the evaluator (e.g., dependent, independent evaluator), and the outcomes measured. Appendix S1 offers a detailed scheme of the variables to be codified. Regarding information about participants that will be coded, we plan to use the labels for ethnicity and gender, or biological sex and gender identity, as reported in the primary studies.

The procedures for searching manuscripts and screening each manuscript for inclusion or exclusion will be meticulously documented. This documentation will be utilised to create a PRISMA flow chart (Liberati et al. 2009) in the final review.

#### 3.3.4 Assessment of Risk of Bias in Included Studies

The risk of quality bias will be assessed using two Joanna Briggs Institute (JBI) structured instruments, each specifically designed to evaluate RCTs and quasi‐experiments.

The Checklist for RCTs evaluates nine critical dimensions to ensure the validity and reliability of RCTs. It entails 13 questions that can be responded to as yes, no, unclear, or not applicable (Tufanaru et al. 2024). The dimensions covered by the checklist are defined as follows:

- *Randomisation and Allocation:* The JBI Checklist for Experimental Studies evaluates whether participants were randomly assigned to treatment groups and if this allocation was concealed, preventing selection bias and ensuring group comparability.
- *Baseline Comparability:* Ensuring baseline similarity of treatment groups is crucial to avoid selection bias. The checklist checks for even distribution of characteristics like age and disease severity, which might otherwise explain the outcomes.
- *Blinding Procedures:* Blinding participants, those delivering the treatment, and outcome assessors prevent biases. This ensures that behaviours and measurements are not influenced by knowledge of group allocation.
- *Treatment Consistency:* The checklist reviews whether treatment groups received identical care except for the intervention. This ensures any observed effects are due to the intervention, not other treatments.
- *Follow‐up and Attrition:* Tracking and reporting follow‐up completeness and differences between groups is essential. Addressing loss to follow‐up prevents bias and clarifies the intervention's effects.
- *Intention‐to‐Treat (ITT) Analysis:* The ITT principle is evaluated to ensure participants are analysed in their original groups, regardless of treatment completion, maintaining the study's integrity.
- *Outcome Measurement:* The checklist ensures outcomes are measured consistently and reliably across groups, ensuring accurate data collection.
- *Statistical Analysis:* Appropriate statistical analysis is critical. The checklist checks if statistical assumptions were met, power analysis was performed, and suitable methods were used.
- *Trial Design Appropriateness:* Finally, the checklist ensures the trial design fits the research topic and justifies any deviations from standard designs, including alternative designs like crossover or cluster RCTs.

The Checklist for Quasi‐Experimental Studies evaluates several critical elements to ensure the validity and reliability of non‐randomised experimental studies (Barker et al. 2024). It entails 9 items that can be responded to as yes, no, unclear, or not applicable. The dimensions covered by the checklist are defined as follows:

- *Clarity of Cause‐and‐Effect Relationship:* The checklist assesses the clarity of the cause‐and‐effect relationship, ensuring there is no ambiguity about the temporal relationship of variables. The independent variable (cause) should precede the dependent variable (effect) in time.
- *Participant Similarity:* It examines the similarity of participants across comparison groups to avoid selection bias, ensuring that differences in outcomes are not due to pre‐existing disparities among participants.
- *Treatment Consistency:* It evaluates whether participants in comparison groups received similar treatments, apart from the intervention of interest, to ensure that observed effects can be attributed solely to the intervention. The presence of a control group is also assessed, as it strengthens causal inferences by providing a basis for comparison against the intervention group.
- *Multiple Measurements and Follow‐up:* The checklist looks for multiple measurements of the outcome both before and after the intervention to establish changes attributable to the intervention and rule out other explanations like natural variations or regression to the mean. The completeness of follow‐up is also scrutinised, with attention to how differences in follow‐up between groups are described and analysed, as incomplete follow‐up can threaten internal validity.
- *Consistency and Reliability of Outcome Measurement:* Consistency in outcome measurement across comparison groups is evaluated to prevent measurement differences from confounding the treatment effects. The reliability of outcome measurements is another crucial aspect, ensuring that measurement methods are consistent and dependable within the study context.
- *Appropriateness of Statistical Analysis:* This includes ensuring that statistical tests respect their assumptions, that there is adequate statistical power, and that the correct statistical methods are applied, given the study's design and objectives.

#### 3.3.5 Measures of Treatment Effect

Effect sizes in this study will be computed following the methodologies recommended by Borenstein et al. (2009) as well as Lipsey and Wilson (2001). For data presented as raw frequencies, percentages, proportions, or rates, odds ratios (ORs) will be employed as the primary metric. These ORs will be initially calculated on a natural log scale to maintain analytical symmetry before being back‐transformed to their original scale for more intuitive interpretation, as advised by Borenstein et al. (2009). All ORs will be reported with 95% confidence intervals: an OR greater than 1 indicates a positive intervention effect, an OR of 1 suggests no effect, and an OR less than 1 points to a negative intervention effect. To ensure consistency across studies, effect sizes may be adjusted so that all outcomes are coded uniformly.

For outcomes reported on continuous scales, the standardised mean difference (SMD) or Cohen's *d* will be calculated, provided that sufficient data are available. In instances where sample sizes are small, the SMD will be corrected to Hedges' *g* using the transformation formula outlined by Lipsey and Wilson (2001), with corresponding 95% confidence intervals provided. When the data set includes a combination of binary and continuous outcomes, the original metrics will be preserved during data collection, with the less common effect sizes being transformed into the more prevalent metric for each outcome.

In cases where expert consultation is necessary, it will be sought to ensure the accurate calculation of effect sizes. Any raw or log‐transformed data will be handled in accordance with the guidelines detailed by Higgins and Green (2011) in the Cochrane Handbook for Systematic Reviews of Interventions. Sensitivity analyses will then be performed to assess the impact of these transformations and ensure the robustness of the findings.

#### 3.3.6 Unit of Analysis

In the present systematic review, both individual participants and groups of individuals (clusters) will be considered as units of analysis when applicable. To ensure proper handling of clustered data, we will adhere to the recommendations provided in the Cochrane Handbook for Systematic Reviews of Interventions (Higgins and Green 2011). Specifically, in cluster‐randomised trials, the effective sample size will be adjusted to prevent the merging of individual and cluster data. This adjustment will be made by dividing the original sample size by the design effect, which is calculated using the formula: 1 + (M − 1) × ICC, where M represents the average cluster size and ICC stands for the intra‐cluster correlation coefficient. This approach ensures that the statistical analysis accurately accounts for the clustered nature of the data.

#### 3.3.7 Criteria for Determination of Independent Findings

The review will include various sources, such as book chapters, journal articles, government reports, and MSc or PhD theses. In instances where the same data is reported in multiple sources (e.g., a journal article and a book chapter), we will code the data only once to prevent overestimation of effect sizes. As per Lipsey and Landenberger (2006), we will prioritise the most frequently reported result across sources, and if necessary, we will select the most comprehensive evaluation record. To address the issue of dependence, we will employ robust variance estimation and a suitable model for meta‐analysis of dependent effects. Given that effect sizes are likely to be correlated within and across studies, a hierarchical model, such as the one recommended by Pustejovsky and Tipton (2022), will be used to account for these dependencies effectively.

#### 3.3.8 Dealing With Missing Data

In accordance with Lipsey and Wilson (2001), efforts will be made to acquire any missing key statistical data by contacting the corresponding authors of the primary studies. Should these attempts prove unsuccessful, the study will be excluded from the effect size synthesis. All studies excluded for this reason will be explicitly identified and systematically reported.

#### 3.3.9 Assessment of Heterogeneity

The study will explore and assess heterogeneity by examining variations in study characteristics, such as the type of intervention, sample populations, and outcome measures, to determine how these factors may influence the effectiveness of interventions aimed at reducing cognitive and behavioural violent radicalisation. Subgroup analyses will be conducted to investigate whether specific characteristics—such as age, gender, geographic location, or the duration and intensity of the intervention—impact the outcomes differently. To statistically test heterogeneity, the *I* ^2^ statistic, *Q* statistic, and *τ* ^2^ (tau‐squared) will be utilised. The *I* ^2^ statistic will quantify the percentage of total variation across studies due to heterogeneity, while the *Q* statistic will assess whether observed differences between studies are due to chance. The *τ* ^2^ statistic will estimate the variance of true effect sizes across studies (Borenstein and Higgins 2013). Any observed heterogeneity will be carefully analysed to understand its sources and their implications for the generalisability of the findings.

#### 3.3.10 Assessment of Reporting Biases

To test publication bias, funnel plots of standard error will be produced. Given that the interpretation of funnel plots can be subjective (e.g., Borenstein et al. 2009), we plan the inclusion of additional statistical tests on the potential publication bias. In this study, we will assess publication bias using Egger's test, a widely used method for detecting bias in meta‐analyses. Specifically, we will apply Egger's test with the sandwich estimator (Egger et al. 1997) to examine whether smaller studies tend to report larger effect sizes, which could suggest the presence of publication bias. The sandwich estimator provides robust standard errors to account for potential heteroscedasticity, ensuring more reliable results in detecting bias.

In addition, we will conduct a moderator analysis to compare the results of published versus unpublished studies and to explore the influence of sample size by comparing studies with small and large sample sizes. This will further help us identify any systematic differences that may arise due to publication bias or the influence of sample size on the effect sizes reported in the studies. Together, these methods will allow for a comprehensive evaluation of potential biases in the data set.

#### 3.3.11 Data Synthesis

To assess the impact of interventions aimed at reducing cognitive and behavioural violent radicalisation, we will perform individual meta‐analytic models to estimate the average effects of interventions on a range of outcomes related to radicalisation and terrorism. To enhance the rigour and accuracy of our analysis, we will utilise a multilevel random‐effects model with robust variance estimation (Borenstein et al. 2010). The multilevel meta‐analytic approach is essential in this context because it accommodates the hierarchical structure of the data, allowing for the inclusion of multiple effect sizes derived from the same study. This is particularly important given that studies on violent radicalisation and terrorism often report multiple outcomes or interventions, leading to non‐independent effect sizes. By accounting for both within‐study and between‐study variation, the multilevel model provides more reliable estimates of the average effect, mitigating potential bias associated with treating non‐independent data points as independent.

Moreover, the use of robust variance estimation ensures that standard errors remain reliable, even when the assumptions of homogeneity and independence are violated, which is particularly pertinent when handling complex data structures. Effect sizes will be weighted inversely by their variances and covariances, a method that appropriately adjusts for the differing levels of precision across studies. This approach is particularly valuable in fields like violent radicalisation and terrorism, where studies often exhibit substantial heterogeneity in both methodological design and sample size. The use of a multilevel model ensures that our analysis captures these variations, providing more generalisable and accurate conclusions regarding the effectiveness of interventions.

The analysis will be conducted using the randomForest library in R‐Studio as well as metafor and clubSandwich (Pustejovsky and Tipton 2022).

#### 3.3.12 Subgroup Analysis and Investigation of Heterogeneity

The study will explore and assess heterogeneity by examining variations in study characteristics, such as the type of intervention, sample populations, and outcome measures, to determine how these factors may influence the effectiveness of interventions aimed at reducing cognitive and behavioural violent radicalisation. Subgroup analyses will be conducted to investigate whether specific characteristics—such as age, gender, geographic location, or the duration and intensity of the intervention—impact the outcomes differently. To statistically test heterogeneity, the *I*² statistic, *Q* statistic, and *τ* ^2^ (tau‐squared) will be utilised. The *I* ^2^ statistic will quantify the percentage of total variation across studies due to heterogeneity, while the *Q* statistic will assess whether observed differences between studies are due to chance. The *τ* ^2^ statistic will estimate the variance of true effect sizes across studies (Borenstein and Higgins 2013). Any observed heterogeneity will be carefully analysed to understand its sources and their implications for the generalisability of the findings.

Given that a sufficient number of studies are retrieved and included, analyses will be performed to explore the potential role of specific moderators (covariates) in explaining the heterogeneity in the results. Based on theory (e.g., Nivette et al. 2017) and previous research (Brouillette‐Alarie et al. 2022; Sydes et al. 2023), several potential effect modifiers have been identified that should be extracted from the selected studies and coded in the data collection instrument (Appendix S1). These moderators will potentially cover the following aspects:

- Participants' characteristics: Moderators such as gender, age, and ethnicity will be tested to explain heterogeneity. Previous research suggests that ethnicity may influence the effectiveness of interventions. Specifically, evidence shows that programmes targeting homogeneous populations (e.g., only Muslims) can exhibit iatrogenic effects (Brouillette‐Alarie et al. 2022). The role of ethnicity and country as moderators of overall effect sizes will be explored.
- Intervention characteristics: When possible, moderators related to the interventions will be tested, such as the type of intervention, duration, quality of implementation, targeted level of prevention, and targeted ideology.
- Theory of change of the interventions: There is an interest in testing whether the theoretical background of interventions (e.g., Cognitive‐Behavioural, Restorative Justice) can moderate the effect of interventions in reducing cognitive and behavioural violent radicalisation/terrorism.
- Quality of the evidence: Factors such as risk of bias, sample size, and study design will be considered.

As mentioned earlier, if the data displays acceptable statistical power, heterogeneity will be explored by running meta‐regression using the ‘metafor’ package (Viechtbauer 2010). If at least five studies compare groups based on moderator variables, analyses will be run under a random‐effects model, using separate estimates of tau‐squared (i.e., variance component) for each group. As previously stated, it is difficult to assume that the true effect between studies is the same for all groups in this review.

#### 3.3.13 Sensitivity Analysis

The study will incorporate sensitivity analyses to rigorously test the robustness of the results and evaluate how different assumptions or methodological choices may influence the overall conclusions. This process will involve conducting alternative analyses by excluding studies with a high risk of bias, such as those with inadequate randomisation, poor blinding, or incomplete outcome data. Additionally, sensitivity analyses will assess the impact of removing smaller studies with limited sample sizes, as these can disproportionately affect the meta‐analysis results. The study will also explore variations in outcome measurement methods by examining how different definitions or assessment tools for key outcomes might influence the findings.

Furthermore, the inclusion or exclusion of unpublished studies, grey literature, or studies with non‐significant results will be tested to assess potential publication bias. By performing these additional analyses, the study aims to determine whether its primary conclusions hold consistently across a range of scenarios or if they are sensitive to specific methodological or data‐driven decisions. This approach will help ensure the reliability and generalisability of the findings, providing a clearer understanding of the intervention's true effectiveness in reducing cognitive and behavioural violent radicalisation.

#### 3.3.14 Treatment of Qualitative Research

Qualitative studies will be excluded from this review.

#### 3.3.15 Summary of Findings and Assessment of the Certainty of the Evidence

We do not plan to include a summary of findings and assessment of the certainty of the evidence.

## Author Contributions

Content: Sara Valdebenito, Manuel Eisner. Systematic review methods: Sara Valdebenito. Statistical analysis: Sara Valdebenito.

## Conflicts of Interest

The authors declare no conflicts of interest.

## Transparent Peer Review

The peer review history for this article is available at https://publons.com/publon/10.1002/cl2.70058.

## Preliminary Timeframe

The approximate date for submission of the systematic review is 1st April 2025.

## Plans for Updating the Review

We plan to produce an updated version of the review every 3 years. The lead author will be in charge of coordinating and producing the revised versions.

## Supporting information

C2‐Protocol terrorism APR2025 Appendices.
