# Supplementary material for: Protocol: Interventions to Prevent Cognitive and Behavioural Violent Radicalisation: A Systematic Review and Multilevel Meta‐Analysis: A Systematic Review
Source: Campbell Syst Rev. 2025 Aug 10;21(3):e70058. doi: 10.1002/cl2.70058 (PMC12335747; doi:10.1002/cl2.70058)
Supplement: Supplementary file 1 — C2‐Protocol terrorism APR2025 Appendices. [file CL2-21-e70058-s001.docx]

## APPENDIX 1: DATA COLLECTION INSTRUMENT

**Interventions to prevent cognitive and behavioural**

**violent radicalisation: A multilevel meta analysis.**

(**adapted from** Valdebenito, Eisner, Farrington, Ttofi and Sutherland, 2018)

**Section A. Bibliographical information**

Before completing this section, please be sure that the manuscript is correctly uploaded in the reference manager programme.

Name of the main author(s):

Year of publication:

Has the paper included a conflict of interest statement?

- 1. Yes
- 0. No

Programme site:

Language of the publication:

- 1. English
- 2. German
- 3. Italian
- 4. Spanish
- 5. Portuguese
- 999. Other (please specify below)

If answer was "999. Other", please specify:

Type of publication:

- 1. Journal
- 2. Unpublished research
- 3. Book/book chapter
- 4. Masters thesis
- 5. PhD/doctoral thesis
- 6. Technical/governmental report
- 7. Conference proceedings
- 999. Other (please specify below)

If answer was "999. Other", please specify:

How was the study/report located?

- 1. Electronic database
- 2. Web search
- 3. Reference in a book/paper (please specify below)
- 4. Hand search in specialised journal
- 5. Peer/expert suggestion
- 999. Other (please specify below)

If answer was "3. Reference in a book/paper", please specify:

If answer was "999. Other", please specify:

**Section B. Ethics**

Did the study declare the use of “consent agreement forms”?

- 1. Yes
- 0. No
- 999. Other (please specify below)
- 99. Unknown

If answer was "999. Other", please specify:

Who signed the consent?

- 1. Participant
- 2. Parents or responsible adult
- 999. Other (please specify below)
- 99. Unknown

If answer was "999. Other", please specify:

**Section C. Design**

The present systematic review includes randomised control trials as well as quasi-experimental reports. Please select always the data that is related with the sample effectively analysed.

What kind of design is this paper based on?

- 1. Randomised controlled trial (true experiment)
- 2. Before-and-after without control/comparison group/s (quasi-experimental)
- 3. Before-and-after with control/comparison group/s (quasi-experimental)
- 4. Propensity score matching (quasi-experimental)
- 5. Interrupted time series (quasi-experimental)
- 6. Pre/post measures with unmatched control/comparison group (quasi-experimental)
- 999. Other (please specify below)

If answer was "999. Other", please specify:

Units of randomisation

- 1. Individuals
- 2. Clusters/groups)
- 999. Other (please specify below)
- 99. Unknown

If answer was "999. Other", please specify:

Unit of analysis

- 1. Individuals
- 2. Clusters/groups
- 999. Other (please specify below)
- 99. Unknown

If answer was "999. Other", please specify:

Variables measured to create comparability? (e.g., variables used to match the control and treatment groups)

What is the main statistical analysis used to produce the final results?

- 1. Multilevel modelling
- 2. Differences of means
- 3. MANOVA
- 4. Chi-squared
- 5. Propensity Score Matching
- 999. Other (please specify below)

If answer was "999. Other", please specify:

Does the study include a post-assessment?

- 1. Yes
- 0. No
- 3. Unclear

If the study includes a post-assessment, how many days after the intervention was the post-assessment carried out?

Was a follow-up included? (after post-test)

- 1. Yes
- 0. No
- 3. Unclear

If follow-up is included, how many times was this carried out?

If any follow-up-assessments were included, how many days after the intervention were these carried out?

What was the response rate for the baseline survey (if applicable)

**Section D. Sample**

How was the sample selected?

- 1. Randomly
- 2. Assessment
- 3. Self-selection
- 999. Other (please specify below)

If answer was "999. Other", please specify:

Inclusion criteria:

Exclusion criteria:

Mean age and standard deviation of overall sample at beginning of intervention:

Population type:

- General
- Youth
- Forensic
- At-risk
- Other
- Unclear

N total:

N of intervention group:

N of control group

Mean age and standard deviation of overall sample at beginning of intervention:

Gender

- Males
- Females
- 99. Unknown

Percentage of male participants

Percentage of female participants

Location of program

- Urban area
- Suburban area
- Rural area
- Mixture of areas
- 99. Not enough information to determine

Predominant ethnicity^^[[1]](#footnote-0)^^

- 1. Caucasian
- 2. Black
- 3. Hispanic
- 4. Asian
- 5. of other mixed background
- 99. Unknown

Percentage of Caucasian participants if known

Percentage of Black participants if known

Percentage of Hispanic participants if known

Percentage of Asian participants if known

Percentage of other mixed background participants if known

Please state the name of the country where sample was located when tested.

What was the ideological background of the majority of the population? (if known)

What was the religious background of the majority of the population? (if known)

**Section E. Programme delivered**

This section aims to codify data on the delivery process. Be aware that sometimes final reports do not describe all the data related to delivery. In those cases it would be helpful to search for registered protocols or earlier publications reporting more data on this.

Name of the programme:

What was the intervention type?

- Mentoring
- Whatsapp
- Digital game
- Sports related
- Other

Intervention description (text+page number):

What was the target audience?

- Individual
- Group

Was the program curricular?

- 1. Yes
- 0. No
- 99. Unknown
- 999. Other (please specify below)

If answer was "999. Other", please specify:

The programme was conducted for:

- 1. Research ends
- 2. Demonstration ends
- 3. Routine
- 99. Unknown
- 999. Other. (please specify below)

If answer was "999. Other", please specify:

Primary programme site:

- 1. Schools
- 2. Community
- 3. Prison
- 4. Probation
- 3. Other (please specify below)
- 99. Unknown

If answer was "999. Other", please specify:

Deliverer’s background 1

- 1. Social worker
- 2. Psychologist
- 3. Teacher
- 4. Police/Probation officers
- 5. Peers
- 999. Other. (please specify below)
- 99. Unknown

If answer was "999. Other", please specify:

Deliverer’s background 2

- 1. Social worker
- 2. Psychologist
- 3. Teacher
- 4. Police officers
- 5. Peers
- 999. Other. (please specify below)
- 99. Unknown

If answer was "999. Other", please specify:

Did the deliverer receive training BEFORE implementing the programme?

- 1. Yes.
- 0. No.
- 99. Unknown

How long was the training in hours?

Did the deliverer receive training DURING the implementation?

- 1. Yes.
- 0. No.
- 99. Unknown

How long was the training in hours?

What type of intervention was delivered?

Theoretical background of the intervention. If the manuscript indicates a mixture of theories, you can select more than one using THEORY 1, 2 and 3.

|  | **[THEORY1]** | **[THEORY2]** | **[THEORY3]** |
| --- | --- | --- | --- |
| 1. Cognitive behavioural |  |  |  |
| 2. Learning theory |  |  |  |
| 3. Restorative theories |  |  |  |
| 4. Deterrance |  |  |  |
| 99. Unknown |  |  |  |
| 999. Other (Specify) |  |  |  |

What happened to the control group?

- 1. No intervention
- 2. Wait-list control
- 3. Minimal contact
- 4. Treatment as usual
- 5. Alternative treatment
- 5. Placebo
- 999. Other. (please specify below)

If answer was "999. Other", please specify:

Delivery format:

- 1. Manualised programme
- 2. Unstructured programme
- 3. Mixed
- 99. Unknown
- 999. Other. (please specify below)

If answer was "999. Other", please specify:

What was the programme dosage?

AVERAGE Duration in weeks:

AVERAGE Hours per week:

What was the frequency of the programme counted?

- 1. Less than a week
- 2. Once a week
- 3. Twice a week
- 4. 3-4 times a week
- 5. Daily
- 99. Unknown

What was the “evaluator” role?

- 1. Deliver the programme
- 2. Designed the programme
- 3. Both design and delivery
- 4. Independent evaluator
- 99. Unknown

Was the programme implementation monitored?

- 1. Yes
- 0. No
- 99. Unknown. Not enough information

Does the report provide information about implementation problems?

- 1. Yes, there were clear problems which are reported
- 0. No, non-reported problems, reasonably well implemented
- 2. Possible problems based on the description of the intervention
- 99. Unknown. Not enough information

Is the cost of the intervention mentioned?

- 1. Yes
- 0. No

Cost:

Currency:

**Section F. Outcome measured and statistical data**

Outcome measured (name of construct):

Outcome definition (as used by authors):

Instrument type

- Continuous
- Dichotomous
- Categorical

Number of items in instrument:

Source of instrument (reference):

Conceptual domain of outcome:

Is ES reported?

- Yes
- No

Standardised?

- Yes
- No

Specify reported ES

If no, can it be calculated based on available data?

- Yes
- No

**Available data in manuscript**

M/SDs

Frequencies or proportions (dicho)

Frequencies or proportions (poly)

Unadjusted corelation coef

Multiple regressions coef (unst)

Multiple regression coef (stand)

OR

T-value

F-value

Chi-square

Other

Statistical adjustment of ES

- Yes
- No

Explanations regarding statistical adjustment of ES

Baseline statistical results (for mean, sd, N)

After intervention results (for mean, sd, N)

Follow-up 1 intervention results (for mean, sd, N)

Follow-up 2intervention results (for mean, sd, N)

Retrospective outcome:

- Yes
- No

Origin outcome:

- Official data
- Self-reported
- Family report
- Practitioner report
- Other

Missing information to be requested from authors:

## APPENDIX 2: SCREENING TOOLS

**Title and abstracts screening**

| **Criteria for Inclusion** | **Yes** | **No** |
| --- | --- | --- |
| **Study Design** |  |  |
| - Uses an experimental or quasi-experimental design to evaluate the impact of interventions aimed at tackling violent radicalisation (RCTs or QEDs) |  |  |
| **Participants** |  |  |
| - Young (i.e., older than 12 years of age) or adult individuals who are at risk of, or involved in, the process of cognitive or behavioural violent radicalisation or acts defined as terrorist. |  |  |
| **Interventions** |  |  |
| - Is the intervention aimed to prevent terrorism and/or violent radicalisation? |  |  |
| **Outcome Measures** |  |  |
| - Reports cognitive or behavioural outcomes related to violent radicalisation |  |  |
| **Publication Type** |  |  |
| - Journal article, book chapter, government report, or academic thesis |  |  |
| **Publication status** |  |  |
| Published or unpublished manuscript |  |  |
| **Language** |  |  |
| - Title and abstract available in English |  |  |
| Final decision: | | |
| Add comments if necessary: | | |

**Full screening**

| **Study Information** | |  |
| --- | --- | --- |
| Study ID:  Title:  Authors:  Publication Year:  Journal/Source: | |  |
| **Inclusion Criteria** | |  |
| **Types of Studies** | Yes | No |
| Study Design: |  |  |
| Uses an experimental or quasi-experimental design to evaluate the impact of interventions aimed at tackling violent radicalisation (RCTs or QEDs) |  |  |
| **Types of Participants** |  |  |
| Target Population:  Young (i.e., older than 12 years of age) or adult individuals who are at risk of, or involved in, the process of cognitive or behavioural violent radicalisation or acts defined as terrorist. |  |  |
| **Types of Interventions** |  |  |
| Intervention Focus:  Does the study focus on interventions targeting individuals at risk of violent radicalisation or those involved in extremist activities? |  |  |
| **Types of Outcome Measures** |  |  |
| Behavioural: Does the study measure changes in terrorist activities, violent behaviours, arrests, convictions, or breaches of parole/probation? |  |  |
| Cognitive: Does the study measure changes in attitudes favouring terrorism, legitimacy perceptions, willingness to join extremist groups, resilience to violent radicalisation, political tolerance, or adherence to civic values? |  |  |
| **Language** |  |  |
| Language of Publication:  Is the title and abstract available in English? |  |  |
| **Exclusion Criteria** |  |  |
| Qualitative Studies:  Does the study use qualitative methods? |  |  |
| Victim Support Interventions:  Does the study focus on interventions for victims of terrorist violence, including children, families, and second responders? |  |  |
| Government Policy Interventions:  Does the study focus on interventions related to government policy changes? |  |  |
| Situational Prevention Strategies:  Does the study test the impact of situational prevention strategies (e.g., metal detectors, walls, street barriers)? |  |  |
| Counter-Narrative and Online Media Campaigns:  Does the study target counter-narrative and online media campaigns? |  |  |
| **Screening Outcome** | |  |
| Final decision:  Exclusion Reason: (if applicable)  Reviewer:  Date: | |  |
| Comments: (if applicable) | |  |

## APPENDIX 3: Core search strategy (APA Psycinfo via ovid)

Platform/Database: APA PsycInfo <1806 to October 2024 Week 5>

Search Date: 11 November 2024

--------------------------------------------------------------------------------

1 extremism/ (940)

2 exp terrorism/ (9278)

3 radical movements/ (453)

4 ("al-qaeda" or "al-qaida" or anarch* or bioterror* or "boko haram" or bomb or bombed or bomber* or bombing* or bombs or cyberterror* or "cyber terror*" or "cyber-terror*" or daesh or extremis* or "extreme-left" or "extreme-right" or "extreme* violen*").ti,ab,id. (6412)

5 (fanatic* or "far-left" or "far-right" or "foreign agent*" or "foreign fighter*" or guerrilla* or hamas or hezbollah or hijack* or incel or incels or indoctrinat* or insurrection* or insurgent* or ISIL or islamis* or jihad* or KKK or "ku klux klan" or klans*).ti,ab,id. (4332)

6 (lethal or lone or loner* or martyr* or militant* or militia* or mujahideen* or neonazi* or "neo-nazi*" or qanon* or radical* or 4chan or 8chan or 8kun).ti,ab,id. (31284)

7 (salafi* or skyjack* or supremacis* or supremacy or sympathiser* or sympathizer* or taliban or terroris* or terror* or violen* or zealot*).ti,ab,id. (130777)

8 or/1-7 (164736)

9 ("free radical*" or "hydroxyl radical*" or "oxygen radical*" or "peroxide radical*" or "radical prostatect*" or "superoxide radical*").ti,ab,id. (2419)

10 8 not 9 (162317)

11 risk management/ (6529)

12 school based intervention/ (24365)

13 interven*.ti,ab,id. (539177)

14 ((alternative* or approach* or "at risk" or "at-risk" or campaign* or communit* or dialog* or diversion* or divert*) adj10 (counter* or deradical* or de-radical* or desist* or disengag* or exit* or prevent* or recidivis* or reduc* or re-enter* or reentry or re-entry or rehab* or reintegrat* or re-integrat*)).ti,ab,id. (69641)

15 ((engage* or engaging or factor* or guidance or implement* or initiative* or interact* or intervene*) adj10 (counter* or deradical* or de-radical* or desist* or disengag* or exit* or prevent* or recidivis* or reduc* or re-enter* or reentry or re-entry or rehab* or reintegrat* or re-integrat*)).ti,ab,id. (84667)

16 ((method* or model* or program* or policy or policies or practice* or project* or scheme* or strateg* or treat*) adj10 (counter* or deradical* or de-radical* or desist* or disengag* or exit* or prevent* or recidivis* or reduc* or re-enter* or reentry or re-entry or rehab* or reintegrat* or re-integrat* or "risk manage*")).ti,ab,id. (268309)

17 ((alternative* or counter* or mitigat* or prevent* or reduc* or rehab* or reintegrat* or re-integrat* or "risk manage*" or stop*) adj5 (extremis* or radical* or terrori*)).ti,ab,id. (1877)

18 or/11-17 (799176)

19 exp randomized controlled trials/ (1682)

20 experiment controls/ (970)

21 placebo/ (6772)

22 (random* or RCT*).ti,ab,id,hw,mf. (271164)

23 (allocat* or assign*).ab. (152820)

24 ((clinical or control*) adj3 trial*).ti,ab,id. (105829)

25 (control* adj3 (experiment* or group*)).ti,ab,id. (133848)

26 (control* adj3 (studies or study)).ti,ab,id. (48893)

27 ((doubl* or singl* or tripl*) adj (blind* or mask*)).ti,ab,id,hw,mf. (38338)

28 (match* adj2 group*).ab. (9826)

29 (nonrandom* or "non random*" or "non-random*").ti,ab,id,hw,mf. (6489)

30 (("open label" or "open-label") adj5 (studies or study or trial*)).ti,ab,id,hw,mf. (5549)

31 placebo*.ti,ab,id,hw,mf. (46985)

32 "pre-post".ti,ab,id,hw,mf. (10227)

33 (quasiexperiment* or "quasi-experiment*" or quasirandom* or "quasi-random*").ti,ab,id,hw,mf. (19129)

34 or/19-33 (551322)

35 10 and 18 and 34 (4142)

36 animal.po. (450489)

37 exp animals/ or animal models/ or exp animal research/ or exp primates/ (400032)

38 or/36-37 (467173)

39 human.po. (4822767)

40 38 not 39 (400710)

41 35 not 40 (4125)

1. Based on Lipsey & Wilson (2001) [↑](#footnote-ref-0)
